# The impact of coping strategies and positive resources on post-traumatic stress symptoms among bereaved families of the Sewol ferry disaster

**DOI:** 10.3389/fpsyt.2024.1367976

**Published:** 2024-04-02

**Authors:** So Hee Lee, Jin-Won Noh, Kyoung-Beom Kim, Jeong-Ho Chae

**Affiliations:** ^1^ Department of Psychiatry, National Medical Center, Seoul, Republic of Korea; ^2^ Division of Health Administration, College of Software and Digital Healthcare Convergence, Yonsei University, Wonju, Republic of Korea; ^3^ Department of International Healthcare Administration, College of Bio and Medical Sciences, Catholic University of Daegu, Daegu, Republic of Korea; ^4^ Department of Psychiatry, Seoul St. Mary’s Hospital, The Catholic University of Korea, College of Medicine, Seoul, Republic of Korea

**Keywords:** Sewol ferry disaster, bereaved families, post-traumatic stress disorder, avoidance coping, optimism

## Abstract

**Introduction:**

This study investigated the long-term prevalence of, and factors associated with, post-traumatic stress disorder (PTSD) among the bereaved families of the Sewol ferry disaster, in which 250 students lost their lives during a school excursion.

**Methods:**

Eight years after the disaster, 181 family members were surveyed, and the prevalence of clinical PTSD symptoms was estimated. The Positive Resources Test (POREST), the Duke-UNC Functional Social Support Questionnaire, and the Brief COPE were evaluated using self-report measures. The multivariable binomial logistic regression was used to identify protective and risk factors for PTSD.

**Results:**

PTSD symptoms were present in 49.7% of the family members 8 years after the incident. A one-point increase in the score on the optimism subscale of the POREST was associated with a 20.1% decreased likelihood of having clinical PTSD symptoms (OR = 0.799; p = 0.027; 95% CI = 0.655–0.975). Conversely, a one-point increase in the score on the avoidant subscale of Brief COPE was associated with a 13.2% increased likelihood of having clinical PTSD symptoms (OR = 1.132; p = 0.041; 95% CI = 1.005–1.274).

**Discussion:**

Our results provide evidence of the need for long-term mental health monitoring of bereaved families of disaster victims, along with valuable insights for the development of mental health intervention programs.

## 1 Introduction

The sinking of the Sewol ferry in 2014 was a tragic incident that claimed the lives of 250 out of the 325 high school students onboard for a field trip. This disaster is regarded as one of South Korea’s worst social catastrophes, sparking extensive criticisms and conflicts related to issues like inadequate ship management, errors in judgment by the captain and crew, delayed response, misleading announcements, and perceived mishandling by the government. Even today, the incident remains a highly sensitive and contentious topic, underscoring the severity of the tragedy and the need to address the systemic failures and shortcomings that led to such a devastating loss of life.

Notably, the families of the deceased students continue to suffer from various mental health issues, including depression, anxiety post-traumatic stress disorder (PTSD), and complicated grief (1–3). After conducting a cross-sectional study on the mental health of Sewol Ferry disaster bereaved families 18 months after the incident, the results revealed that 94% experienced complicated grief, 50% reported severe depression, and 70% exhibited clinically significant post-traumatic symptoms (PTSS) (2). When assessing the embitterment of the accident-affected families, it was found to be 63% at 18 months post-incident, increasing to 77% at 30 months. The group experiencing an increase also showed a concurrent rise in anxiety, post-traumatic stress symptoms, and complicated grief (3). Furthermore, the group diagnosed with PTSD and complicated grief among the accident-affected families was found to be associated with a perception of injustice, according to the analysis results (1). Understanding the factors related to PTSD might be crucial to improve these disaster-bereaved families’ long-term mental health.

Numerous studies have made significant efforts to identify the risk and protective factors for PTSD (4–10). Coping strategies have been found to play a vital role in mental health outcomes, particularly in individuals who have experienced trauma (4). Adaptive coping strategies often lead to positive outcomes, while maladaptive strategies, such as substance use, may lead to greater impairment (5). Additionally, social support has been identified as a protective factor of PTSD. In a study investigating the impact of a tornado on adolescents, social support, extent of tornado exposure, and sex significantly influenced the development of PTSD (6). Moreover, a meta-analysis study on the influence of social support on PTSS in children and adolescents revealed that most longitudinal studies have indicated that social support is a significant predictor of PTSS (7, 11). According to the results of an online survey conducted among medical students who were locked down during the COVID-19 pandemic period, social support mediated the relationship between positive coping and post-traumatic stress symptoms (8). Furthermore, positive expectancies, self-efficacy, optimism, and hope have been associated with less severe PTSD symptoms. The results of a meta-analysis indicated that positive expectations were predictive of less severe PTSD symptoms (9). Additionally, hope and positivity were associated with post-traumatic growth in oral cancer patients (10).

The burden of PTSD following disasters is known to be significant, and it is associated with various factors such as sociodemographic and background factors, event exposure characteristics, social support factors, and personality traits (12). One of the longitudinal studies on the long-term PTSD symptoms among disaster bereaved families is the research related to the 2011 Utøya terror attack in Norway (13). According to this study, eight years after the disaster, many bereaved parents and siblings were showing long-lasting health consequences with symptoms of PG (Prolonged Grief) and PTS (Post-Traumatic Stress) as well as functional impairment. Moreover, the results of a study conducted 26 years after the 1990 fire on the Scandinavian Star ferry found that high social support plays a significant role in reducing posttraumatic stress symptoms, particularly among individuals with a ruminative coping style (14). In addition, when investigating counterfactual thinking among them, it was suggested that vivid counterfactuals about a traumatic event play a role similar to trauma memories in post-traumatic stress, indicating that they are not beneficial (15).

While various studies have been conducted on the mental health of disaster-bereaved families, there has been relatively limited research on long-term PTSD risk factors and protective factors following disasters. Therefore, this study investigated the plausible predictive role of coping strategies, social support, and positive resources in the long-term prognosis of PTSD in the families of the victims of the Sewol ferry disaster. The study intends to provide valuable evidence that can serve as a foundation for developing mental health-promoting programs tailored to families affected by disasters.

## 2 Materials and methods

### 2.1 Study sample and design

This study was conducted in 2022, eight years after the Sewol ferry disaster, targeting parents who lost their children in the accident. With the assistance of the Ansan Onmaeum Center, we met with representatives of bereaved families to explain the purpose and procedures of this study. The representatives understood the intention of our study and promoted it online to the bereaved families, encouraging those interested to participate by sending messages via their mobile phones. Over a period of approximately two months, the research team scheduled individual appointments and conducted surveys with the participants. The analysis included data from 181 participants after excluding 21 individuals with incomplete responses.

#### 2.1.1 Outcome variables

PTSD symptoms were assessed using the PTSD Checklist for DSM-5 (PCL-5), based on the fifth edition of the Diagnostic and Statistical Manual of Mental Disorders (16). We ensured that participants responded to PTSD symptoms related to the disaster by adding the phrase ‘related to the Sewol ferry incident.’. The PCL-5 comprised 20 items, and total scores range from 0 to 80. A total score of 33 indicated a provisional diagnosis of PTSD. The Korean version of the PCL-5 was utilized in this study, and it demonstrated good internal consistency and test-retest reliability in the present study (Cronbach’s alpha coefficient of 0.963) (17).

#### 2.1.2 Independent variables

In the current study, positive psychological resources were assessed using the Positive Resources Test (POREST), a self-reported instrument for assessing optimism, purpose/hope, self-control, social support, and care (18). The POREST consists of 23 items, and participants provide their responses on a five-point Likert scale ranging from 1 (not true) to 5 (very true). Total scores on the POREST range from 23 to 115, with higher scores indicating more personal positive resources. The Cronbach’s alpha coefficient for the POREST in this study was 0.919, indicating good internal consistency.

Social support was measured using the Duke-UNC Functional Social Support Questionnaire (FSSQ) (19). The Korean version of the FSSQ has been validated, demonstrating good psychometric properties (20). The FSSQ comprises 14 items, and participants rate their responses on a five-point Likert scale ranging from 1 (much less than I would like) to 5 (as much as I would like). Total scores on the FSSQ range from 14 to 70, with higher scores indicating higher levels of perceived social support. The Cronbach’s alpha coefficient for the FSSQ in this study was 0.954, indicating high internal consistency.

Coping strategies were assessed using the Brief COPE, a well-established self-report measure developed by Carver (1997). The Brief COPE was used to assess coping responses to stress and challenging situations. It comprises 28 items that assess coping strategies commonly employed in response to stressors. The Brief COPE assesses three coping categories: problem-focused coping (active coping, planning, and instrumental support), emotion-focused coping (positive reframing, humor, religion, acceptance, and emotional support), and avoidant coping (self-blame, behavioral engagement, substance abuse, self-distraction, denial, and venting). Each item is rated on a four-point Likert scale. Higher scores indicate greater utilization of stress coping strategies. The Cronbach’s alpha coefficient for the Brief COPE in this study was 0.835, indicating good internal consistency.

#### 2.1.3 Covariates

The analyses were adjusted for several covariates, including age, sex, type of health insurance, marital status, and household income. Age was a numeric variable, while sex was dichotomized into male and female categories. The type of health insurance was classified into two groups: national health insurance and “others”. The others category included individuals who were beneficiaries of the medical aid program and those who refused to answer. The South Korean medical aid program is comparable to the Medicaid program in the United States, and its beneficiaries include individuals with low socioeconomic status (21). Marital status was dichotomized as married and “others” (those who were separated, divorced, widowed, or had never been married). Household income was categorized into four groups: ≤ 1.99, 2.00–3.99, ≥ 4.00, and those who refused to answer. Household income was measured in monthly units of 1 million South Korean Won, approximately equivalent to USD 1,265 as of 2022.

### 2.2 Analytical approach and statistics

Descriptive analysis was conducted to summarize the baseline characteristics of the participants and clinically classify the PTSD symptoms. The results are presented as mean and standard deviation (SD) for normally distributed numerical data, median and interquartile range (IQR) for non-normally distributed numerical data, and frequency and percentages for categorical data, as appropriate. To assess the relationship based on the clinical classification of PTSD symptoms, the two-sample t-test was used for analyzing normally distributed numerical data, the two-sample Wilcoxon rank-sum test was used for analyzing non-normally distributed numerical data, and Fisher’s exact test was used to analyze the categorical data. Binomial logistic regression was performed to investigate the relationships between related factors for PTSD while controlling for covariates. To assess multicollinearity in the multivariable regression model, the variance inflation factor (VIF) was calculated. Huber-White’s sandwich estimator was employed to calculate the heteroscedasticity-robust standard error (22). The threshold for statistical significance was set at p < 0.05 for two-tailed tests. All statistical analyses were conducted using Stata/MP 17.0 software (Stata Corp., College Station, TX, USA).

## 3 Results

**Table 1** presents a summary of the baseline characteristics and clinical classifications of PTSD for the study participants. The study included 181 participants with a median age of 53 years, and 56.9% of the participants were female. The proportion of participants exhibiting PTSD symptoms that required clinical attention was 49.7% (n = 90). Among the non-clinical PTSD group, the mean or median score for the FSSQ, and the total score for the POREST and its subscales (except the care subscale), were significantly higher. However, the mean score for the avoidant subscale of the Brief COPE was significantly higher in the clinical PTSD group. Additionally, household income showed a significant negative association with clinical PTSD symptoms. The mean PCL-5 score of study participants were 32.6 ± 19.7.

**Table 1:** Clinical classification and baseline characteristics of bereaved family members of the Sewol ferry disaster.

Variable	Post-traumatic stress disorder (PCL-5)						
	Non-clinical group (n = 91; 50.3%)		Clinical group (n = 90; 49.7%)		Total (n = 181)		*p-value*
	n	r%	n	r%	n	c%	
Age (min=26; max=68) ^*^ **^†^**	54	7	53	6	53	6	0.486
Sex							
Male	41	52.6	37	47.4	78	43.1	0.653
Female	50	48.5	53	51.5	103	56.9	
Type of healthcare insurance							
NHI	80	50.3	79	49.7	159	87.8	0.999
Medical aid	11	50.0	11	50.0	22	12.2	
Marital status							
Married	82	52.6	74	47.4	156	86.2	0.137
Others	9	36.0	16	64.0	25	13.8	
Household income (per million KRW; monthly)							
1.99 or below	9	37.5	15	62.5	24	13.3	0.003
Between 2.00 and 3.99	22	46.8	25	53.2	47	26.0	
4.00 or above	36	72.0	14	28.0	50	27.6	
Not answered	24	40.0	36	60.0	60	33.1	
Positive resources (POREST)							
Total score (min=28; max=106) **^†^**	67	21	57	23	64	22.5	0.001
Optimism (min=7; max=32) ^*^	19.1	5.0	15.6	4.8	17.2	5.2	< 0.001
Purpose/hope (min=6; max=30) ^*^	16.4	5.6	14.6	5.2	15.4	5.5	0.044
Self-control (min=5; max=24) ^*^	14.7	4.0	13.2	3.9	13.9	4.0	0.024
Social support (min=3; max=15) **^†^**	9	3	8	4	8	4	0.001
Care (min=2; max=10) ^*^	6.7	1.9	6.3	2.1	6.5	2.0	0.154
Social support (FSSQ) (min=14; max=70) ^*^	44.9	14.2	37.4	13.6	41.8	14.4	0.004
Coping strategy (Brief COPE)							
Total score (min=28; max=90) ^*^	55.9	10.5	57.6	10.1	56.8	10.3	0.329
Problem focused (min=6; max=22) **^†^**	13	5	12	6	12	5	0.244
Emotion focused (min=10; max=33) **^†^**	19	5	17	8	18	6	0.114
Avoidant (min=12; max=42) ^*^	24.3	5.9	27.8	5.7	26.1	6.1	< 0.001

^*^ Values presented as mean and standard deviation (SD).

^†^ Values presented as median and interquartile range (IQR).Clinical subgroup comparisons were performed using Fisher’s exact tests for categorical data, two-sample t-test for normally distributed numerical data, and two-sample Wilcoxon rank-sum test for non-normally distributed numerical data.

r%, row percentage; c%, column percentage; NHI, National Health Insurance; KRW, South Korean Won (one million KRW ≈ 1265 US Dollar); PTSD, Post-traumatic stress disorder; PCL-5, PTSD Checklist for DSM-5 (the fifth edition of the Diagnostic and Statistical Manual of Mental Disorders); POREST, Positive Resources Test; FSSQ, Functional Social Support Questionnaire; Brief COPE, Brief Coping Orientation to Problems Experienced.

**Table 2** presents the results of the multivariable binomial logistic regression, which aimed to identify protective and risk factors for PTSD. A one-point increase in the score on the optimism subscale of the POREST was associated with a 20.1% decreased likelihood of having clinical PTSD symptoms (OR = 0.799; p = 0.027; 95% CI = 0.655–0.975). Conversely, a one-point increase in the score on the avoidant subscale of Brief COPE was associated with a 13.2% increased likelihood of having clinical PTSD symptoms (OR = 1.132; p = 0.041; 95% CI = 1.005–1.274).

**Table 2:** Protective and Risk Factors associated with post-traumatic stress disorder.

Variable	OR	Robust SE	*p*-value	LL	UL
Age (min=26; max=68)	1.117	0.100	0.216	0.937	1.331
Sex					
Male	ref				
Female	1.837	1.253	0.373	0.483	6.990
Type of healthcare insurance					
NHI	ref				
Medical aid	0.473	0.513	0.490	0.056	3.971
Marital status					
Married	ref				
Others	6.562	8.314	0.138	0.548	78.606
Monthly Household income (per million KRW; monthly)					
1.99 or below	ref				
Between 2.00 and 3.99	1.943	1.940	0.506	0.274	13.752
4.00 or above	1.000	0.954	0.999	0.154	6.490
Not answered	1.670	1.548	0.580	0.272	10.268
Positive resources (POREST)					
Optimism (min=7; max=32)	0.799	0.081	0.027	0.655	0.975
Purpose/hope (min=6; max=30)	1.093	0.112	0.382	0.895	1.336
Self-control (min=5; max=24)	0.949	0.088	0.570	0.791	1.138
Social support (min=3; max=15)	0.740	0.128	0.082	0.527	1.039
Care (min=2; max=10)	0.827	0.224	0.483	0.487	1.406
Social support (FSSQ) (min=14; max=70)	1.019	0.031	0.548	0.959	1.082
Coping strategy (Brief COPE)					
Problem focused (min=6; max=22)	0.997	0.122	0.981	0.785	1.267
Emotion focused (min=10; max=33)	1.180	0.138	0.155	0.939	1.483
Avoidant (min=12; max=42)	1.132	0.069	0.041	1.005	1.274

OR, odds ratio; SE, standard error; CI, confidence interval; LL, lower limit of 95% CI; UL, upper limit of 95% CI, ref, reference group; NHI, National Health Insurance; KRW, South Korean Won (one million KRW ≈ 1265 US Dollar); PTSD, Post-traumatic stress disorder; PCL-5, PTSD Checklist for DSM-5 (the fifth edition of the Diagnostic and Statistical Manual of Mental Disorders); POREST, Positive Resources Test; FSSQ, Functional Social Support Questionnaire; Brief COPE, Brief Coping Orientation to Problems Experienced.

## 4 Discussion

In this study, we tracked the mental health of the Sewol ferry disaster victims’ families and found that, 8 years after the incident, 49.7% of the families had clinically significant PTSD symptoms. The use of avoidance coping strategies was identified as a risk factor for PTSD, while optimism was a protective factor.

These results have important implications. First, they highlight the lasting impact of traumatic events on mental health, with the prevalence of PTSD being significantly higher in our sample compared to families affected by other disasters in previous studies. For example, families who experienced the 2004 tsunami disaster had a PTSD prevalence of 34.5%, while bereaved survivors of the 2008 Sichuan earthquake had a prevalence of 16.8% at 18 months after the event (23, 24). The higher prevalence in the Sewol ferry case may be attributable to the nature of the incident, which was a tragic accident during a school excursion (rather than a natural disaster) potentially worsened by the sense of preventability and the role of human error. A systematic literature review on PTSD following man-made disasters reported PTSD prevalence rates ranging from approximately 20% to 75% among survivors or rescuers (12). Bereaved families from the 2011 Utøya terror attack in Norway still showed clinical levels of PTSS in approximately 46% of cases eight years after the disaster (13). While there may be differences in results depending on the research methodology, the prevalence of PTSD tends to be lower following natural disasters compared to human-made and technological disasters (12). Additionally, it should be considered in the interpretation of the results that the participants of this study are parents who have lost children in disasters. According to the review study, bereaved parents exhibited a higher prevalence of mental health issues compared to bereaved spouses and parents who have not experienced loss (25). Furthermore, the study’s focus on PTSD symptoms 8 years after the disaster emphasizes the long-term nature of mental health consequences. This underscores the importance of continued monitoring and support for affected families over extended periods, as trauma-related symptoms may manifest or change over time.

Second, the identification of avoidance coping strategies as a risk factor for PTSD among bereaved families aligns with the existing research on maladaptive coping in response to trauma. A meta-analysis revealed that high occupational stress and avoidant coping strategies significantly increase the risk of PTSD among police officers (26). This finding underscores the importance of addressing and modifying avoidance behaviors in therapeutic interventions to promote healthier coping mechanisms and better mental health outcomes for traumatized individuals.

Third, optimism was found to be a protective factor against PTSD, which is encouraging. Previous studies have demonstrated that positive expectancies, such as hope, self-efficacy, and optimism, act as protective factors against PTSD (9, 10). In our study, we specifically found that among the positive resources including optimism, hope, self-control, social support and care, optimism was significantly associated with less severe PTSD symptoms. This suggests that, in the process of striving for nearly a decade to uncover the truth of the tragedy of the Sewol ferry accident, where parents of the victims lost their children, the factor of optimism would be beneficial to their mental health by alleviating feelings of injustice and resentment. This implies that fostering a sense of optimism could play a crucial role in promoting resilience and reducing the impact of trauma on mental health. Integrating optimism-focused interventions into mental health programs may prove beneficial for families affected by disasters.

Fourth, it was noteworthy that this study did not find evidence that social support influences PTSD in the bereaved families of the Sewol ferry disaster. This result contrast with previous research findings in disaster-experienced individuals, suggesting that social support may be one of the protective factors for PTSD (6–8). The bereaved parents have reported experiencing profound embitterment after losing their children in the human-made ferry accident, going through feelings of being cheated, injustice, incompetence, wrongdoing by a perpetrator, and the destruction of their belief and value system (27). In a qualitative analysis of interviews with Sewol ferry disaster bereaved families, it was revealed that they are maintaining only minimal interpersonal relationships due to social withdrawal (28). They reported difficulties trusting others and expressed caution when someone tries to get closer to them since the incident. Survivors who directly witnessed the 2016 attacks in Belgium reported experiencing changes such as aggression, guilt, distrust, or psychosomatic factors like migraine attacks after the incident (29). They felt that others would not understand them, leading to a deterioration in interpersonal relationships. In that regard, it has been suggested that for the improvement of PTSD symptoms, it is important to make clients feel safe in therapy settings and that therapeutic relationships play a role similar to social support (30). Furthermore, the socio-ecological model of resilience has been proposed, suggesting that the best care for trauma survivors is not limited to assisting the individual alone but is achievable when a combination of interpersonal relationships and societal services they belong to is provided. There have been longitudinal studies on the relationship between social support and PTSS depending on the timing after a disaster. Initially after the disaster, it was possible to explain the relationship through social causation (more social support leading to less PTSD), whereas later on, it was found that social selection (more PTSD leading to less social support) was the operative mechanism (31). Therefore, it can be interpreted that, perhaps, bereaved families have experienced significant negative impacts on interpersonal relationships after the incident. As a result, the positive effects of social support on mental health may have been negligible.

The study had several limitations. First, the limited sample, which comprised only 181 families out of an estimated 500 bereaved parents of the 250 deceased students, may impact the generalizability of the findings. Participant recruitment was challenging given the families’ anger and skepticism after the accident, which limited the sample size. Nonetheless, the fact that about half of the families registered for the study engaged in long-term follow-up surveys was significant. Second, the method of participant recruitment may have introduced sampling bias, potentially affecting the representativeness of the target population. Therefore, there may be differences between the participating families and those who did not participate. Participants in the study may be more adversely affected by the negative impacts of the incident compared to non-participants. Third, the cross-sectional nature of the study may limit the ability to establish causality between variables, as associations were analyzed at a specific time point. Fourth, even though participants were asked to respond to symptoms related to the Sewol ferry disaster when evaluating PTSD, it is not possible to completely exclude the influence of other events and incidents over the span of eight years. In this regard, given the significantly high prevalence of PTSD among Sewol ferry bereaved parents, follow-up longitudinal studies are necessary.

Despite its limitations, this study provides valuable insights into the mental health challenges faced by parents who lost their children in a human-made disaster and the factors influencing long-term PTSD. The results have practical implications for mental health practitioners and policymakers involved in designing interventions for disaster survivors and victims’ families.

## Data availability statement

The datasets presented in this article are not readily available because we did not obtain permission from the subjects to disclose the dataset. Requests to access the datasets should be directed to So Hee Lee, sohee.lee@nmc.or.kr.

## Ethics statement

The studies involving humans were approved by the Institutional Review Board of the National Medical Center (registration No. NMC-2022-07-079). The studies were conducted in accordance with the local legislation and institutional requirements. The participants provided their written informed consent to participate in this study.

## Author contributions

SL: Conceptualization, Funding acquisition, Project administration, Supervision, Writing – original draft, Resources, Writing – review & editing. JN: Data curation, Formal analysis, Methodology, Software, Validation, Writing – original draft. KK: Formal analysis, Investigation, Methodology, Resources, Software, Writing – original draft, Visualization. JC: Project administration, Writing – review & editing, Conceptualization, Supervision, Validation.
